# Application of modified π-shaped anastomosis in digestive tract reconstruction during totally laparoscopic total gastrectomy: a feasibility study

**DOI:** 10.1186/s12957-026-04332-4

**Published:** 2026-04-11

**Authors:** Yinghu Hu, Senlin Wan, Changming Liang, Zhenyuan Li, Mengyun Wang, Xu Zhang, Lishuai Xu, Fangshuai Hu, Dawei Zhang, Yongchun Liu, Xiaoming Wang, Yabin Xia, Xiaoxu Huang, Li Xu

**Affiliations:** 1 Department of Gastrointestinal Surgery, Yijishan Hospital of Wannan Medical College, Wuhu, Anhui China; 2 Department of Gastrointestinal Surgery, Ren Ji Hospital, Shanghai Jiao Tong University School of Medicine, No. 2000, Jiangyue Road, Shanghai, 200000 China; 3 Department of Gastrointestinal Surgery, The Second Affiliated Hospital of Wannan Medical College, No. 10, Rehabilitation Road, Wuhu, Anhui 241000 China

**Keywords:** Totally laparoscopic total gastrectomy, Gastrointestinal reconstruction, Modified π-shaped anastomosis

## Abstract

**Objective:**

To investigate the feasibility and short-term efficacy of the modified π-shaped anastomosis technique for esophagogastric anastomosis in gastrointestinal reconstruction after total gastrectomy performed entirely by laparoscopy.

**Methods:**

This study included 50 patients who underwent totally laparoscopic gastrectomy with modified π-shaped anastomosis for gastrointestinal reconstruction at the First Affiliated Hospital of Wannan Medical College from August 2021 to August 2023. In this study, intraoperative and postoperative indicators were measured to assess the feasibility and short-term efficacy of this technique. Our modified surgical approach involved first resecting the jejunal mesentery and dividing the jejunum during gastrointestinal tract reconstruction, followed by esophagojejunal anastomosis.

**Results:**

The surgery was successfully completed for all 50 patients. The operation time was 251.3 ± 43.2 min, the postoperative hospital stay was 9.3 ± 1.3 days, the time to first postoperative ventilation from the end of the surgery was 4.3 ± 1.9 days, and the time to first postoperative feeding was 7.8 ± 1.6. No complications such as intestinal obstruction, anastomotic fistula, or anastomotic stenosis were observed. One patient developed anastomotic bleeding, one patient experienced an incisional infection, and two patients had pleural effusions. All 50 patients were followed up for more than one year, with no evidence of long-term complications or recurrence.

**Conclusion:**

Modified π-shaped anastomosis is effective for digestive tract reconstruction during totally laparoscopic total gastrectomy (TLTG), thereby effectively preventing mucosal eversion, reducing anastomotic tension, and decreasing the incidence of anastomotic leakage.

## Introduction

Globally, gastric cancer currently ranks fifth in incidence and fourth in mortality among all malignant tumors. More alarmingly, more than 970,000 new cases of gastric cancer are diagnosed annually worldwide, accounting for 4.9% of all new cancer cases [1–3]. In recent years, the overall global incidence of gastric cancer has declined, largely because of *Helicobacter pylori* prevention and control efforts, as well as the widespread use of refrigerators to reduce the consumption of pickled foods. However, the incidence of cardia cancer has increased in some developed countries, such as the United States, and is potentially linked to gastroesophageal reflux disease and obesity [4, 5].

Currently, surgical intervention remains the primary treatment modality. Recent advancements in surgical techniques have increasingly favored minimally invasive approaches, with laparoscopic surgery experiencing rapid development. Totally laparoscopic total gastrectomy (TLTG) has emerged as one of the mainstream surgical procedures for treating gastric cancer in recent years and is characterized by minimal trauma, clear surgical visualization, and low postoperative complication rates. Esophagogastroduodenal reconstruction constitutes a critical step in the process of restoring gastrointestinal continuity following total gastrectomy [6–8]. No consensus regarding the optimal technique for gastrointestinal reconstruction in TLTG has yet been reached. The most widely adopted anastomotic methods are π anastomosis and overlap anastomosis [9, 10].

In 2016, Korean scholars first reported π-shaped anastomosis, named for its resemblance to the mathematical symbol π. This technique uses a linear stapler and has been reported to simplify procedures compared with conventional laparoscopic anastomoses, reducing the number of steps while offering high safety and fewer postoperative complications [10–12]. However, the π-shaped structure of the anastomotic site may increase the risk of intestinal loop adhesions. For patients with insufficient esophageal resection margins, severe obesity, or short mesentery, there is currently insufficient evidence to confirm the safety of this approach [8, 13, 14]. In this study, modified π-shaped anastomosis was employed for all intraoperative gastrointestinal reconstructions to achieve proper mesenteric resection and ensure adequate blood supply and low mesenteric tension at the esophagogastric anastomosis, thereby guaranteeing anastomotic quality. The safety and feasibility of this modified π-type anastomosis technique in gastrointestinal reconstruction during TLTG was investigated in this study, with the aim of providing a reference for the selection of anastomotic methods in future clinical gastrointestinal reconstructive procedures.

## Methods

### Study design

In this retrospective study, 50 patients who underwent TLTG at the First Affiliated Hospital of Wannan Medical College between August 2021 and August 2023 were analyzed. Written informed consent was obtained preoperatively, and we explained the advantages and disadvantages of this procedure to patients prior to surgery. Institutional review board approval was secured before initiating the study. All patients were included in the one-year post-operative follow-up. This study was conducted in accordance with the principles outlined in the Declaration of Helsinki. This study was approved by the Ethics Committee of the First Affiliated Hospital of Wannan Medical College and registered with the China Clinical Trial Registry (ChiCTR2300075919). All surgical procedures were performed by a gastrointestinal surgical team comprising one chief surgeon, two attending surgeons, and one resident surgeon. All of the surgeons involved in this study have extensive experience in laparoscopic surgery and gastric cancer resection procedures. The chief surgeon has 15 years of experience in independent practice in gastrointestinal oncology and performed 300 laparoscopic gastrectomies prior to participating in this study. All surgeons adhered to a standardized surgical protocol to ensure consistency in surgical technique and perioperative management.

### Inclusion and exclusion criteria

The inclusion criteria were as follows: (1) patients aged 18–75 years; (2) patients with a gastric malignancy confirmed by endoscopy and pathology, with a tumor located in the gastric body, subcardia, or fundus, and patients with resectable gastric cancer; (3) patients that received no prior treatment before surgery; and (4) patients that had an American Society of Anesthesiologists (ASA) physical status of grade I–III. The exclusion criteria were as follows: (1) patients with two or more cancers or cancers causing obstruction, perforation, or other conditions requiring urgent surgery; (2) patients requiring concomitant resection of adjacent organs; (3) patients with severe hypertension, diabetes, or other underlying conditions rendering them unable to tolerate surgery; (4) patients with extensive intra-abdominal adhesions due to multiple prior abdominal surgeries; (5) patients who refused to sign the informed consent form; and (6) patients whose surgeries were performed by surgeons lacking prior experience in TLTG.

### Surgical methodology

All procedures were performed by the same surgical team. Informed consent forms were reviewed and signed. Preoperative preparations included completing relevant examinations, performing skin preparation and blood transfusion readiness, optimizing systemic conditions, administering oral antibiotics for bowel preparation, and performing gastrointestinal decompression when necessary. Following stable induction of general anesthesia, endotracheal intubation was performed to secure the airway. The patient was placed in a supine position with the arms fixed at the sides. Standard sterile draping and preparation were implemented. A 1.2 cm skin incision was made along the umbilicus. A Veress needle was used to establish CO₂ pneumoperitoneum, with the intra-abdominal pressure set at 12–14 mmHg (1.60–1.86 kPa).

**Fig. 1:**
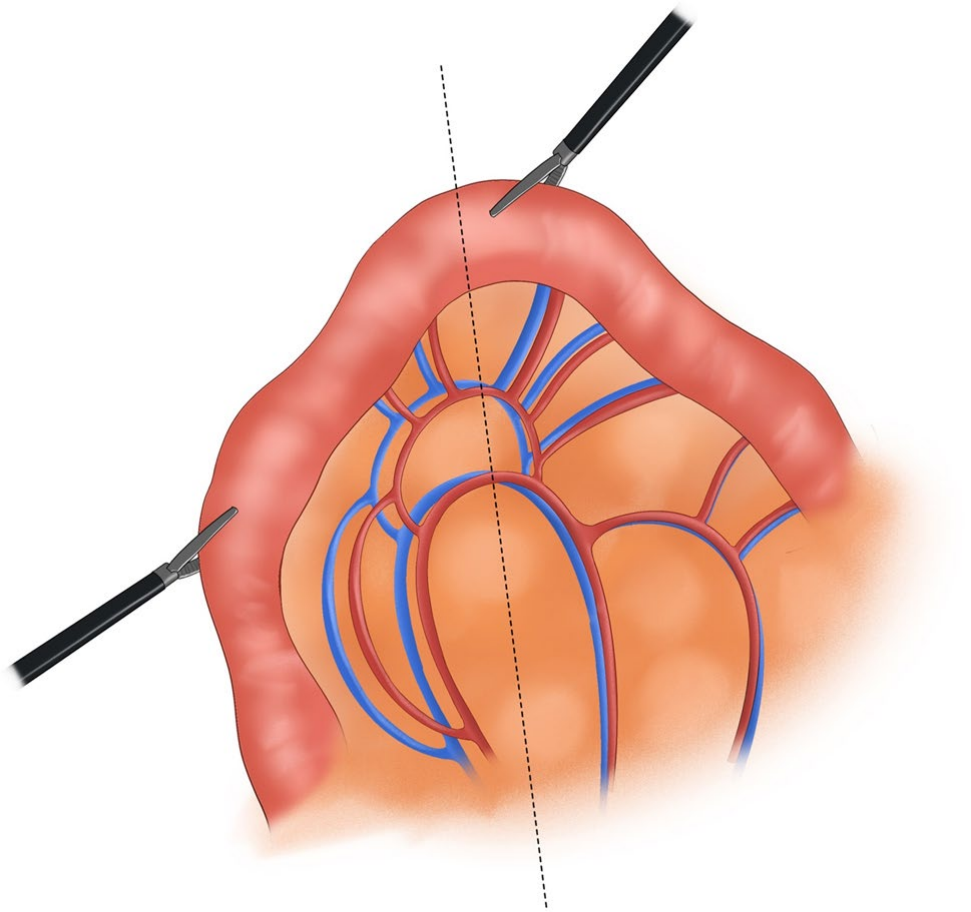
Mobilize the jejunal mesentery

**Fig. 2:**
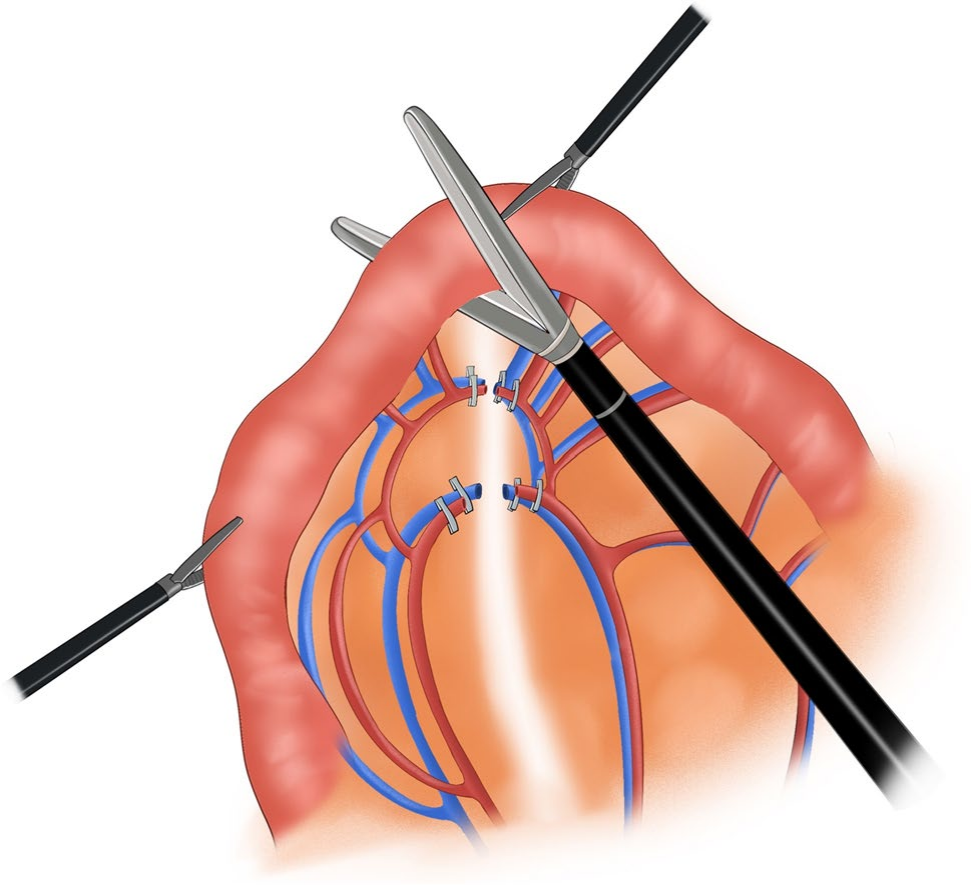
Transect the jejunum

**Fig. 3:**
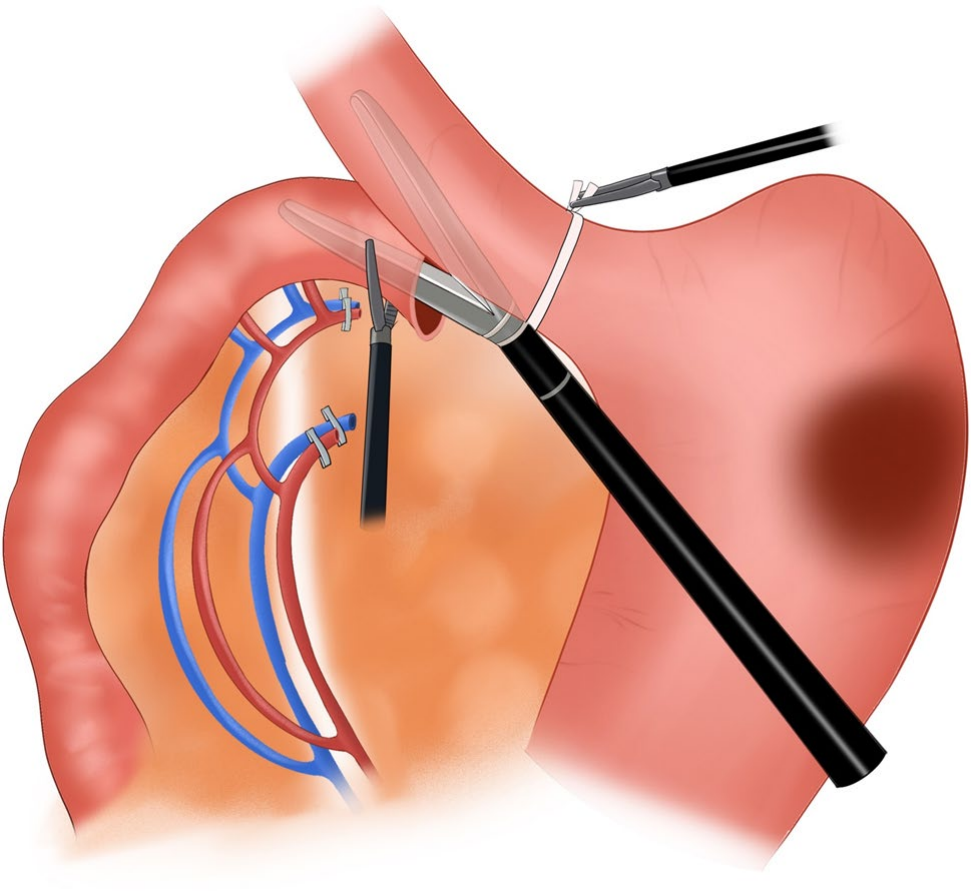
Esophagojejunal anastomosis

**Fig. 4:**
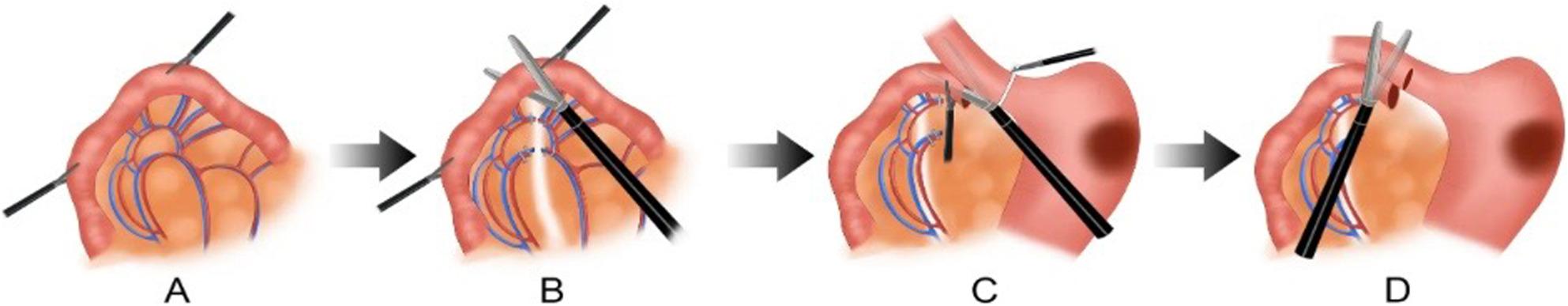
Surgical diagram for modified π-shaped anastomosis; **A** Mobilize the jejunal mesentery; **B** Transect the jejunum; **C** Esophagojejunal anastomosis; **D** Close the common enterotomy

Subsequently, 0.5 cm trocars were placed at the right paracentral position and 6 cm from the right paramedian position near the umbilical area, and a 1.1 cm trocar was placed at the left paramedian position. Laparoscopes and corresponding equipment were inserted through these trocars. The liver, spleen, pancreas, pelvis, transverse colon, and mesentery were intraoperatively examined to confirm that there was no tumor metastasis. The tumor location, size, and distance from the dentate line were assessed to determine the surgical approach. The pyloric region of the stomach was thoroughly dissected down to 3 cm below the pylorus. A linear cutting stapler was used to transect the duodenum 2 cm below the pylorus, concurrently clearing the surrounding lymph nodes. The duodenal stump was wrapped with 3–0 barbed sutures. A suitable preanastomotic bowel was identified 20 cm from the Treitz ligament. With assisted support, the corresponding jejunal mesentery was fully retracted (Fig. 1). An appropriate approach was selected on the basis of the course of the jejunal artery and corresponding vascular arch branches. In obese patients, the jejunal mesentery is typically thicker; in such cases, an ultrasonic scalpel was used to dissect the mesentery layer by layer from a position slightly below the midline between the intestinal wall and the mesenteric root. The corresponding jejunal artery was exposed, ligated, and transected. The mesentery along the jejunal artery–vascular arch–intestinal wall direction was dissected, and care was taken to avoid damaging the arterial arch. If mesenteric tension remained high after ligating and transecting one jejunal artery, a second adjacent jejunal artery was transected using the same method, provided that adequate blood supply to the jejunal segment was maintained. Afterward, a linear stapler was inserted to transect the jejunum (Fig. 2). Without transecting the esophagus, the esophagus was ligated and retracted downward using a ligature. A longitudinal incision was made in the anterior wall of the esophagus and the distal jejunal stump. The linear cutter was positioned at the corresponding sites in the lower esophagus and distal jejunum, the incision was closed, and the linear cutter was activated to perform esophagojejunal side-to-side anastomosis (Fig. 3). The linear cutter was placed and activated 1–2 cm distal to the anastomosis to close the common opening of the esophagus and jejunum, completing total gastrectomy. The linear cutter was positioned as close to the patient’s head as possible to obtain more esophageal tissue and ensure negative margins. A linear stapler was placed 50 cm distal to the Treitz ligament, and a side-to-side anastomosis with the proximal jejunum was performed. The common opening of the esophagus and jejunum was closed with a linear stapler. After intestinal reconstruction was completed, the jejunal mesentery was closed with 3–0 barbed sutures. A 5 cm midline incision was made around the umbilicus. The entire gastric specimen was removed through the incision. After the absence of active bleeding was confirmed, the abdominal cavity was irrigated, and thorough hemostasis was achieved. One latex drainage tube was placed at the esophagogastric anastomosis site, exiting through a right upper quadrant puncture site. Another drainage tube was placed at the splenic fossa, exiting through a left upper quadrant puncture site. Position two additional tubes at the pelvic floor, securing each with sutures to the skin for monitoring potential leaks and bleeding. The abdominal cavity layers were closed sequentially using continuous suturing techniques (Fig. 4).

### Statistical analysis

The data were analyzed using SPSS version 26.0 software. Continuous variables are presented as the mean ± standard deviation. Categorical variables are expressed as frequencies (percentages).

## Results

### Patient characteristics

This was a retrospective study of 50 patients who underwent TLTG at the First Affiliated Hospital of Wannan Medical College from August 2021 to August 2023. All 50 procedures were successfully completed under a fully laparoscopic approach. The relevant clinical data of 37 male patients and 13 female patients are detailed in Table 1. The median age and body mass index (BMI) were 64.9 ± 8.7 and 19.6 ± 2.7, respectively. All patients underwent preoperative ASA physical status classification: 3 patients (6%) had an ASA grade of 1, 43 patients (86%) had an ASA grade of 2, and 4 patients (8%) had an ASA grade of 3 [15]. The mean tumor size was 2.6 ± 1.3 cm. Six patients (12%) were diagnosed with body cancer, 36 patients (72%) with fundic cancer, and 8 patients (16%) with cardia cancer. In terms of TNM stage, 20 patients (40%) had stage I disease, 26 patients (52%) had stage II disease, and 4 patients (8%) had stage III disease [16]. Postoperative histological differentiation revealed poorly differentiated adenocarcinoma as the most common type (30 patients, 60%), followed by moderately differentiated adenocarcinoma (16 patients, 32%). Well-differentiated adenocarcinoma was observed in 2 patients (4%), undifferentiated adenocarcinoma in 1 patient (2%), and signet ring cell carcinoma in 1 patient (2%) [17] (Table 1).

**Table 1:** Characteristics of all patients

Variable		Value
Age(year)		64.9 ± 8.7
Gender	Man	37(74%)
	Female	13(26%)
BMI(kg/m²)		19.6 ± 2.7
ASA	Ⅰ	3(6%)
	Ⅱ	43(86%)
	Ⅲ	4(8%)
Tumor size (cm)		2.6 ± 1.3
Tumor location	Gastric body	6(12%)
	Gastric fundus	36(72%)
	Cardia	8(16%)
Clinical staging	Ⅰ	20(40%)
	ⅡA	14(28%)
	ⅡB	12(24%)
	Ⅲ	4(8%)
Pathological classification	Low-grade adenocarcinoma	30(60%)
	Medullary adenocarcinoma	16(32%)
	Highly differentiated adenocarcinoma	2(4%)
	mucinous adenocarcinoma	1(2%)
	signet ring cell carcinoma	1(2%)

Data are presented as n (%), or mean±standard deviation

*BMI* Body mass index, *ASA* American Society of Anesthesiologists

### Surgical characteristics

The mean operative time was 251.3 ± 43.2 min. Intraoperative blood loss (as measured by the suction volume) was 61.8 ± 43.5 ml. All 50 patients received intraoperative blood transfusions. The mean number of lymph nodes removed (as determined by postoperative pathology) was 29.8 ± 3.2, with a mean number of positive lymph nodes of 10.5 ± 2.1.

### Postoperative recovery and complications

The median time to first postoperative ventilation from the end of the surgery was 4.3 ± 1.9 days, the time to first postoperative feeding was 7.8 ± 1.6 days, the postoperative hospital stay was 9.3 ± 1.3 days, and the time to drainage tube removal was 7.3 ± 1.8 days. One patient developed an anastomotic fistula and presented with early symptoms, including abdominal pain and low-grade fever. After symptomatic management with antimicrobial therapy and drainage tube irrigation, the patient was discharged safely. Two patients developed pleural effusion, which was considered to have been caused by prolonged malnutrition and hypoalbuminemia. One patient developed an incisional infection. After suture removal, it was determined to be caused by subcutaneous fat liquefaction. This patient had a long history of diabetes with poor glycemic control and significant blood glucose fluctuations. No postoperative complications, such as anastomotic bleeding, anastomotic stricture, or pulmonary infection, occurred. All patients were followed up for one year post-operatively, with no deaths reported.

## Discussion

In recent years, the demand for minimally invasive procedures and advances in endoscopic technology have driven surgeons to pursue greater safety and minimal invasiveness without compromising surgical quality. Totally laparoscopic total gastrectomy (TLTG) has become a mainstream approach for gastric cancer treatment, in which esophagojejunal anastomosis is a critical step that directly impacts postoperative oral intake and nutritional status [18, 19]. This center emphasizes that appropriate resection of the jejunal mesentery is essential to ensure anastomotic quality. Excessive resection increases tension on the mesentery and may damage the jejunal vascular arch, compromising the blood supply and leading to complications such as intestinal necrosis or anastomotic leakage [19, 20].

The jejunum is supplied by branches of the superior mesenteric artery, which form vascular arches within the mesentery before giving off straight arteries to the intestinal wall [18]. During surgery, a segment about 20 cm from the Treitz ligament is identified. The mesentery is carefully unfolded, and a dissection path is planned according to the vascular anatomy. In obese patients with a thicker mesentery, an ultrasonic scalpel is used to dissect layer by layer, starting slightly below the midpoint between the bowel wall and the mesenteric root. The target jejunal artery is ligated and divided, followed by dissection toward the vascular arch and intestinal wall, preserving the arch whenever possible. If tension remains, an adjacent jejunal artery may also be divided while maintaining adequate perfusion to the jejunal segment.

Currently, the π-shaped and overlap anastomoses are the most commonly used techniques for esophagojejunal reconstruction, each with distinct technical and clinical profiles [19, 21]. Overlap anastomosis is a peristaltic-oriented method performed with a linear stapler. After transecting the esophagus and jejunum, barbed sutures are placed to approximate the ends before completing the anastomosis [20]. Although technically demanding, it promotes physiological function, reduces reflux, preserves blood supply, and reduces the risks of leakage and stricture.

In contrast, the π-shaped anastomosis employs a linear cutter inserted through a longitudinal esophageal incision to create a side-to-side anastomosis on the right side of the esophagus, where space is more ample. In other words, the π-shaped anastomosis technique involves inserting a linear cutter through a longitudinal esophageal incision to perform a side-to-side anastomosis on the more spacious right side of the esophagus [20]. The key factor here is the tension in the mesentery after esophagogastric anastomosis. Before creating the jejunal opening, the jejunum can be elevated from a point 20–30 cm distal to the Treitz ligament, passing anterior to the transverse colon, to the distal esophagus. This allows for preliminary assessment of the tension at the esophagogastric anastomosis site. Some scholars suggest that direct esophagogastric anastomosis without dissecting the mesentery generally avoids tension issues [22]. However, in clinical practice, the author has observed that for certain patients—particularly those who are obese or have a short mesentery—direct anastomosis without dissecting the mesentery can result in excessive tension, increasing the risk of anastomotic leakage [23, 24]. Therefore, π anastomosis is not recommended for patients with a short mesentery. If significant tension exists during anastomosis, teams with limited proficiency in fully laparoscopic techniques may face the risk of perforating the jejunum to complete the anastomosis [25].

This retrospective study included 50 patients undergoing TLTG with a modified π-shaped anastomosis. Unlike the conventional technique, we first partially divided the jejunal mesentery while preserving the vascular supply, then mobilized and transected the bowel. The distal jejunum was elevated to the anastomotic plane to assess the tension, and a tension-free esophagojejunal anastomosis was performed using a linear cutting stapler, which provides a wider anastomotic lumen than circular staplers and may reduce the risk of stricture and leakage. To maintain exposure, the esophagus was retracted with a ligature during the anastomosis to prevent stump retraction into the chest, which could increase the surgical difficulty or cause mediastinal infection [26, 27].

The mean operative time in this study was 251.3 ± 43.2 min, slightly longer than reported in other series, largely due to the time spent on meticulous jejunal mesentery resection. However, intraoperative blood loss, lymph node yield, time to flatus and oral intake, and hospital stay were comparable to previous studies, supporting the feasibility of this approach. Margin safety is crucial in oncologic surgery. Since the anastomosis is completed before obtaining margins, if tumor proximity is suspected during esophageal dissection, the resection margin must be adjusted; intraoperative frozen sections can be used when necessary [28–30].

Our experience suggests that the modified π-shaped anastomosis is most suitable for patients with gastric cancer located in the body, subcardia, or fundus, with stage II or III disease. For tumors at the gastroesophageal junction, this technique may compromise the determination of the esophageal resection plane: a higher plane shortens the esophageal stump and increases the tension, while a lower plane risks positive margins [31, 32]. Therefore, careful preoperative patient selection is essential for successful implementation (Table 2).

**Table 2:** Comparison of original π anastomosis and modified π anastomosis

Variable	Original π Anastomosis	Modified π Anastomosis
Time of esophagogastric anastomosis	Average shorter	Average longer
Anastomotic tension	More tension	Free tension
Anastomotic blood supply	Abundant	Abundant
Difficulty of Learning Surgical Procedures	Easy	Easier

The modified π-shaped technique evolved from the conventional π-anastomosis based on clinical evaluation. All 50 patients were followed for one year without major long-term complications. However, this study has several limitations: it is a single-center retrospective analysis; the average BMI was low, reflecting the typical profile of gastric cancer patients; the sample size was small, and larger multi-surgeon studies are needed; and the one-year follow-up is insufficient to evaluate long-term oncological outcomes.

## Conclusion

In summary, the present study has preliminarily confirmed the good clinical feasibility and safety of the modified π-shaped anastomosis for gastrointestinal reconstruction after gastrectomy. To further verify these study outcomes, a prospective comparative study with rigorous design is required to directly compare the modified π-shaped anastomosis with the original one in subsequent research.

## Data Availability

No datasets were generated or analysed during the current study.
